# Evolution of Superinfection Immunity in Cluster A Mycobacteriophages

**DOI:** 10.1128/mBio.00971-19

**Published:** 2019-06-04

**Authors:** Travis N. Mavrich, Graham F. Hatfull

**Affiliations:** a Department of Biological Sciences, University of Pittsburgh, Pittsburgh, Pennsylvania, USA; Carnegie Mellon University; Ohio State University; IST Austria; UCSF

**Keywords:** bacteriophage evolution, bacteriophage genetics, bacteriophages

## Abstract

Temperate phages encode an immunity system to control lytic gene expression during lysogeny. This gene regulatory circuit consists of multiple interacting genetic elements, and although it is essential for controlling phage growth, it is subject to conflicting evolutionary pressures. During superinfection of a lysogen, the prophage’s circuit interacts with the superinfecting phage’s circuit and prevents lytic growth if the two circuits are closely related. The circuitry is advantageous since it provides the prophage with a defense mechanism, but the circuitry is also disadvantageous since it limits the phage’s host range during superinfection. Evolutionarily related phages have divergent, orthogonal immunity systems that no longer interact and are heteroimmune, but we do not understand how immunity systems evolve new specificities. Here, we use a group of Cluster A mycobacteriophages that exhibit a spectrum of genetic diversity to examine how immunity system evolution impacts superinfection immunity. We show that phages with mesotypic (i.e., genetically related but distinct) immunity systems exhibit asymmetric and incomplete superinfection phenotypes. They form complex immunity networks instead of well-defined immunity groups, and mutations conferring escape (i.e., virulence) from homotypic or mesotypic immunity have various escape specificities. Thus, virulence and the evolution of new immune specificities are shaped by interactions with homotypic and mesotypic immunity systems.

## Abstract (precis)

Many aspects regarding superinfection, immunity, virulence, and the evolution of immune specificities are poorly understood due to the lack of large collections of isolated and sequenced phages with a spectrum of genetic diversity. Using a genetically diverse collection of Cluster A phages, we show that the classical and relatively straightforward patterns of homoimmunity, heteroimmunity, and virulence result from interactions between homotypic and heterotypic phages at the extreme edges of an evolutionary continuum of immune specificities. Genetic interactions between mesotypic phages result in more complex mesoimmunity phenotypes and virulence profiles. These results highlight that the evolution of immune specificities can be shaped by homotypic and mesotypic interactions and may be more dynamic than previously considered.

## INTRODUCTION

**FIG 1:**
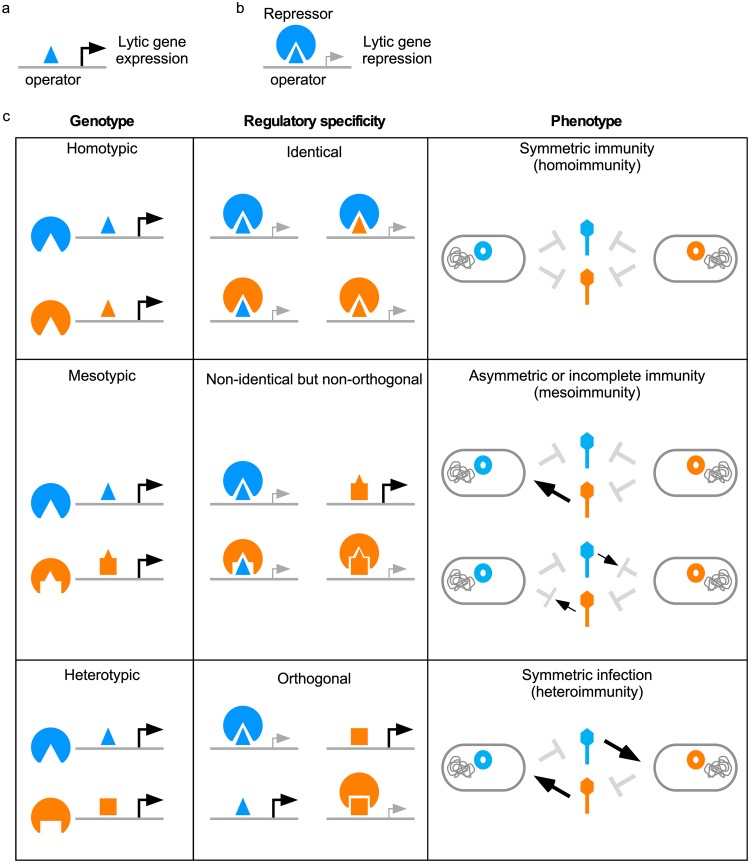
Relationship between superinfection immunity system genotypes and phenotypes. (a and b) Temperate phages contain gene regulatory elements, such as a repressor (circle) and cognate operator sites (triangles), that form a regulatory circuit with distinct specificities to control the expression of lytic genes. (c) During the process of superinfection, the resident prophage and challenging phage may contain homotypic (identical or nearly identical) genetic elements with identical specificities that result in symmetric immunity phenotypes (homoimmunity). Alternatively, they may contain heterotypic (unrelated) immunity systems, exhibiting orthogonal specificities that do not genetically interact, resulting in symmetric infection phenotypes (heteroimmunity). In these cases, the common outcome of superinfection is a binary phenotype (complete infection or complete immunity). However, they may also contain mesotypic (i.e., genetically related but distinct) genetic elements, exhibiting nonidentical but nonorthogonal specificities that still form some genetic interactions, resulting in asymmetric or nonbinary, incomplete immunity phenotypes (mesoimmunity).

Bacteriophages have been in an evolutionary arms race for billions of years against not only the bacterial hosts that they infect but also other bacteriophages that are competing for the same resources (1, 2). Many phages are temperate and can choose between lytic or lysogenic life cycles (3). Although lysogeny may be evolutionarily beneficial, the host remains susceptible to a second round of infection by a genetic spectrum of other phages that are closely related (homotypic), moderately related (here referred to as “mesotypic”), or unrelated (heterotypic) to the resident prophage (Fig. 1) (4–6). As a result, temperate phages must evolve mechanisms to control lysogeny while also defending against other superinfecting phages and escaping other prophage defenses.

Phage immunity systems, critical components of the temperate life cycle, are a target of these evolutionary forces. Immunity systems are genetic circuits that control the temporal expression of genes required for lytic growth (Fig. 1). Coliphage λ harbors the most highly characterized system, a single genetic locus comprised of two DNA-binding transcriptional regulatory genes, *cI* and *cro*, that compete for binding of two tripartite operator sites (7). During lysogeny, CI binds to the operators to block transcription initiation of *cro*, which is required for lytic growth. Immunity systems are diverse and vary in complexity, sometimes consisting of multiple genetic loci (7–9). Coliphage P22 contains a bipartite immunity system, in which the C2 transcriptional repressor performs a function analogous to that of λ CI but is regulated by a second locus, *immI*, from which the Mnt repressor and Ant antirepressor are expressed (10). The tripartite immunity systems in coliphages P1, P7, and N15 are even more complex, utilizing multiple transcriptional regulators expressed from three genetic loci (such as *immC*, *immI*, and *immT* in P1) to create multilayered circuits (8, 11).

The immunity system is required to maintain lysogeny, but it also impacts the process of superinfection. Homoimmune coliphages HK97 and λ harbor homotypic immunity systems, and a λ prophage confers immunity to the host against superinfection from both phages since CI can recognize their lytic gene regulatory elements and prevent lytic growth (Fig. 1) (12). As a result of these genetic interactions, superinfecting phages can escape homotypic immunity by acquiring mutations that disrupt this circuitry (8, 13, 14). λ requires at least three point mutations within operators to superinfect a λ lysogen, as the prophage-expressed CI is unable to recognize the mutant operators and prevent lytic gene expression (7). Additionally, phages that harbor evolutionarily diverged, heterotypic derivatives of the same regulatory circuitry are no longer subject to each other’s circuitry (Fig. 1) (7, 8, 15, 16). Heteroimmune coliphages 434 and λ harbor homologous circuitry, but their CI repressors exhibit specificity for different operator sequences and are unable to block *cro* expression in the opposing phage (7, 17).

The evolutionary process in which homotypic immunity systems diverge and develop distinct heterotypic specificities is poorly understood. In general, superinfection homoimmunity and heteroimmunity are simple symmetric binary phenotypes, in which reciprocal prophage-phage interactions produce the same phenotype of either complete defense or a complete absence of defense (Fig. 1). However, these likely reflect extreme relationships encountered when comparing a small number of individual phages. Although repressor DNA-binding recognition can be mutationally altered with a small number of amino acid substitutions (18, 19), immune specificity itself involves multiple regulatory elements, including secondary immunity loci, multiple operators, and additional phage-encoded proteins, and thus, switching of immune specificities is unlikely to occur in a single mutational step (7). However, if multiple mutational events are required, the process of immune specificity evolution will involve transitional stages in which immunity is incomplete and may be associated with either increased or decreased susceptibility to other phages with related immunity systems. Thus, natural communities of phages are likely to include not only closely related (homotypic) and unrelated (heterotypic) temperate phages exhibiting homoimmunity and heteroimmunity but also phages that are moderately related but distinct (mesotypic), with intermediate immune specificities (here referred to as “mesoimmunity”) (Fig. 1).

A large collection of sequenced mycobacteriophages isolated through the Science Education Alliance Phage Hunters Advancing Genomics and Evolutionary Science (SEA-PHAGES) program provides an opportunity to explore these immune relationships among naturally occurring phages and gain insights into how immune specificity evolves (https://phagesdb.org) (20). Mycobacteriophages are diverse and can be sorted into groups of related types (Clusters A, B, and C, etc.) based on sequence similarity, gene content, and synteny (16, 21–23). Cluster A is the largest and contains over 300 temperate phages, which are subdivided into 19 subclusters (Subclusters A1, A2, and A3, etc.); all have similar genomic architectures but encompass substantial variation.

**FIG 2:**
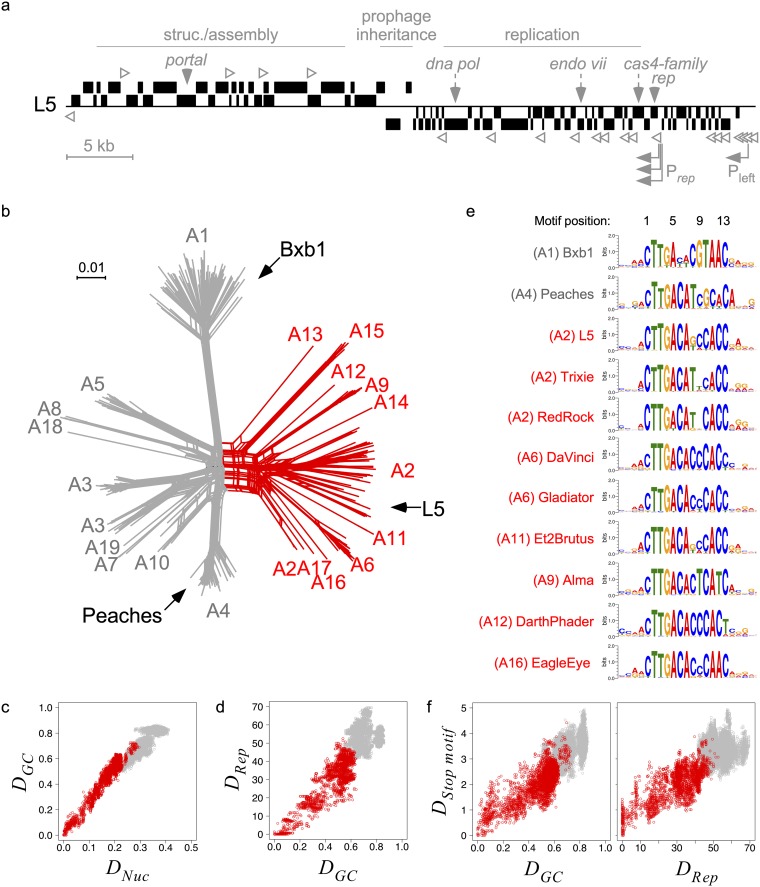
Immunity systems of L5 clade phages exhibit a genetic spectrum. (a) Genome map of L5, with several features highlighted. Genes (black boxes) positioned above or below the line indicate transcriptional orientation. Many genes associated with virion structure and assembly are positioned in the left arm, many genes associated with replication are positioned in the right arm, and genes associated with prophage inheritance (integrase or *parABS*) are positioned in the center. The positions of several genes highly conserved among Cluster A phages are indicated (*dna pol*, DNA polymerase; *endo vii*, endonuclease VII, *rep*, immunity repressor). The early lytic promoter, P_left_, and multiple repressor promoters, P*_rep_*, are indicated by arrows, and asymmetric stoperators are indicated by open arrowheads. (b) Phylogenetic network of 311 Cluster A phages based on gene content using Splitstree. Groups of taxa are labeled with their subcluster designation, several phages are labeled for reference, and a clade of phages representing 10 subclusters that are more closely related to L5 than others are highlighted in red. The number of mycobacteriophages in the L5 clade is 87. (c) Scatterplot comparing whole-genome nucleotide (*D_Nuc_*) and gene content (*D_GC_*) distances involving one Cluster A mycobacteriophage within the L5 clade and another Cluster A mycobacteriophage within (red) or without (gray) the L5 clade. (d) Scatterplot comparing pairwise whole-genome gene content (*D_GC_*) and Rep (*D_Rep_*) genetic distances between Cluster A phages, as described above for panel c. (e) Alignment of sequence motif logos representing predicted stoperator sites for several phages within the L5 clade (red) compared to heterotypic phages Bxb1 and Peaches (gray), with subclusters indicated (see Materials and Methods). (f) Scatterplot comparing pairwise whole-genome gene content (*D_GC_*) or Rep (*D_Rep_*) genetic distances with stoperator motif distances (*D_Stop motif_*) between phages, as described above for panel c. *D_Nuc_* ranges from 0 (100% sequence similarity) to 0.5 (no sequence similarity). *D_GC_* ranges from 0 (all phams are identical) to 1 (no phams are identical). *D_Rep_* ranges from 0 to 100 amino acid substitutions per 100 amino acids. *D_Stop motif_* equals zero for identical motifs, and larger values reflect higher degrees of dissimilarity. See Materials and Methods for further description of distance measurements.

The best-characterized Cluster A immune systems of phages L5 (Subcluster A2) and Bxb1 (Subcluster A1) differ from the λ system in several ways. Each genome encodes a single immunity repressor (Rep) analogous to CI, but there is no evidence for a Cro analog (9, 24) or the divergent transcription of CI and Cro that is common to many temperate phages, including other mycobacteriophages (25). Rep may dimerize in solution, but it binds as a monomer to 13- to 14-bp sites lacking dyad symmetry, and no cooperativity of DNA binding is observed (9, 26, 27). Furthermore, there are 20 to 30 repressor binding sites located throughout the genome oriented with the direction of transcription (Fig. 2a) (9, 24, 28). Some of these sites are bona fide operators located within promoters, such as the P_left_ early lytic promoter. However, most sites are not promoter associated, and the repressor binds these “stoperators” to block transcription elongation (9, 29). L5 and Bxb1 typify the relationships between phages of different subclusters in that they are heteroimmune, their stoperators/operators have distinct consensus sequences, and their repressors show strong binding preferences for their cognate binding sites (26).

Here, we take advantage of the large and diverse group of Cluster A phages to investigate their evolving immune specificities. In addition to homoimmunity and heteroimmunity, we find a surprisingly broad spectrum of mesoimmune specificities, including partial (or incomplete) and asymmetric superinfection immunities (Fig. 1). The variety of immunity phenotypes provides a rich landscape for mutational variation with push-pull dynamics, moving toward either shared immunity or escape from immunity. The evolutionary trajectories are thus likely to be nonlinear as they navigate a complex spectrum of phage relationships.

## RESULTS

### Characterization of the Cluster A immunity system.

All Cluster A phages, including L5, exhibit similar genomic architectures (Fig. 2a). The left arm contains structural and assembly genes, the right arm contains genes associated with lytic growth such as DNA replication, and the genome center contains prophage inheritance genes such as integration or partitioning systems (16, 30). The immunity repressor can be readily identified at syntenic positions, and the early lytic promoter, P_left_, is near the right genome terminus (Fig. 2a). Despite the conserved synteny, these phages are genetically diverse and have been further subdivided into 19 subclusters (Fig. 2b). Phages from distinct subclusters, such as Bxb1 (Subcluster A1), L5 (Subcluster A2), and Peaches (Subcluster A4), have highly divergent repressors and stoperator motifs and are heteroimmune (16). However, the genetic diversity within and between subclusters is not homogenous, and there is a clade of nearly 100 phages representing 10 subclusters that are more closely related to L5 than Bxb1 or Peaches (Fig. 2b). All phages in this “L5 clade” (except for those in Subcluster A15) infect Mycobacterium smegmatis mc^2^155, and they exhibit a spectrum of genetic diversity based on their gene content and nucleotide sequence (Fig. 2c). We therefore focused on the superinfection immunity relationships of phages in this clade, beginning with analysis of the sequence relationships and their divergence.

Immunity repressors in the L5 clade are similar in size (see Fig. S1a in the supplemental material) and exhibit a genetic spectrum that correlates with whole-genome gene content distances (Fig. 2d). As seen with L5, Bxb1, and Peaches, a set of stoperator sites can be identified in each genome (see Materials and Methods). Similar numbers of stoperators are present in each genome, and they predominantly exhibit one orientation relative to the direction of transcription (Fig. S1b and c) (9, 26). Sequence motifs representing each genome’s cognate stoperators are similar, but not identical, to each other (Fig. 2e), and they exhibit a genetic spectrum that also correlates with whole-genome gene content distances as well as repressor distances (Fig. 2f).

10.1128/mBio.00971-19.1 FIG S1 Characterization of Cluster A immunity systems and prophage expression patterns. (a) Box plot of immunity repressor amino acid sizes from 82 L5 clade phages. (b) Histogram of the number of L5 clade phage genomes that contain the indicated number of stoperator sites. (c) Scatterplot comparing the percentages of stoperator sites positioned in the right or left arm of the genome that are oriented in the direction of transcription (syn oriented) for all L5 clade phages (see Materials and Methods). The *y *= *x* line is plotted for reference. (d) Strand-specific RNA-seq expression profiles (*y* axes reflect the number of reads × 1,000) for top (T) and bottom (B) strands of Et2Brutus (Subcluster A11), Gladiator (Subcluster A6), and Trixie (Subcluster A2) phages during lysogeny. Specific loci of interest are highlighted. (e) Histogram reflecting the distribution of stoperator sites identified in all L5 clade phages relative to the right genome termini. Download FIG S1, TIF file, 0.5 MB. Copyright © 2019 Mavrich and Hatfull. 2019 Mavrich and Hatfull This content is distributed under the terms of the Creative Commons Attribution 4.0 International license.

### Gene expression profiles.

The genetic diversity of phages in the L5 clade raises questions as to whether they exhibit similar expression profiles and whether they carry additional genetic elements that may interfere with superinfection, such as secondary immunity loci or prophage-mediated defense systems. Transcriptome sequencing (RNA-seq) analysis of Et2Brutus (Subcluster A11), Gladiator (Subcluster A6), and Trixie (Subcluster A2) lysogens (Fig. S1d) showed patterns similar to those reported previously for Cluster A phages StarStuff, L5, RedRock, Alma, EagleEye, and Pioneer (30, 31), with the only genes expressed other than the repressor being the integrase or *parABS* (30) loci. We observed no evidence of secondary immunity loci or other defense systems similar to those reported for other phages (1, 32).

**FIG 3:**
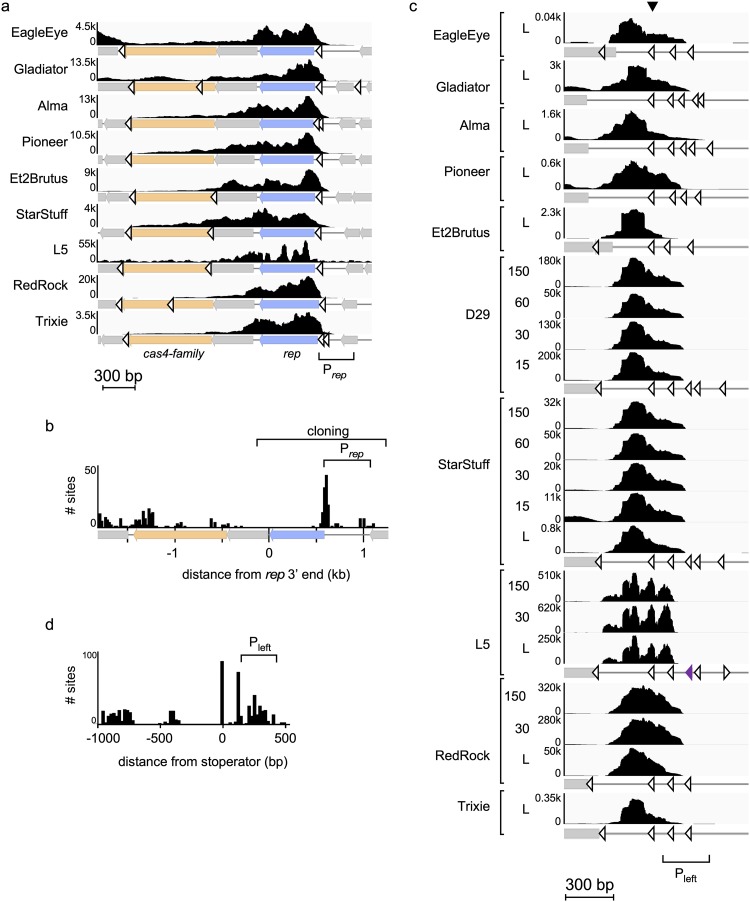
L5 clade phages exhibit similar immunity system architectures. (a) Enlarged view of bottom-strand expression profiles (*y* axes reflect the number of reads × 1,000) across the repressor locus for phages from Fig. S1d in the supplemental material as well as for several previously reported Cluster A phages (30, 31) during lysogeny, with the *rep* (blue) gene, the highly conserved *cas4*-family (orange) gene, stoperators (open arrowheads), and the region containing the empirically determined *rep* promoter indicated. (b) Histogram reflecting the distribution of stoperator sites at the *rep* locus in all L5 clade phages, aligned by the 3′ end of *rep*, with a generic gene map below. The region cloned from several phages to test for repressor-mediated immunity is indicated. (c) Enlarged view of bottom-strand expression profiles (*y* axes reflect the number of reads × 1,000) at the P_left_ locus for phages from Fig. S1d as well as for several previously reported Cluster A phages during lysogeny (L) and lytic growth (15, 30, 60, or 150 min postinfection). Gray boxes, genes; open arrowheads, predicted stoperators; purple arrowhead, empirically identified L5 operator. Genomes are manually aligned by the last predicted stoperator site upstream of the highly expressed region (black arrowhead). (d) Histogram reflecting the distribution of stoperator sites at the P_left_ locus in all L5 clade phages aligned by the same stoperator as in panel c.

In each of these phages, repressor expression initiates in the upstream intergenic region, extends across *rep*, and substantially decreases across the adjacent gene of unknown function and the Cas4-family gene (Fig. 3a). A conserved repressor binding site is located in this intergenic region, which in L5 is proposed to be involved in autoregulation of repressor synthesis (Fig. 3a and b) (28). One or more stoperator sites are located in the downstream genes and may act to reduce the level of transcription (Fig. 3a and b). We observed some expression at the right end of the genomes near P_left_ (Fig. S1d), which likely results from low levels of spontaneous lytic induction rather than lysogenic expression *per se* (31). This region is very highly expressed in early lytic growth (Fig. 3c), and the presence of multiple repressor binding sites, including at least one operator site at the P_left_ promoter, downregulates this region during lysogeny (Fig. 3d and Fig. S1e) (9).

### Rep_Trixie_ discriminates between stoperators of different L5 clade phages.

Although the stoperator motifs of these phages are similar, they also vary at several positions, which may correspond to different repressor binding specificities (Fig. 2e). To demonstrate this, we overexpressed and purified Rep_Trixie_ (Fig. S2a) and determined its binding affinities for a syntenic site in several phages (Fig. S2b). Rep_Trixie_ has the strongest affinity for Trixie and RedRock stoperators (equilibrium dissociation constant [*K_D_*] of ∼6 to 7 nM, which is comparable to that of Rep_L5_ and Rep_Bxb1_ [9, 26]), a somewhat reduced affinity for a Gladiator stoperator, and a very low affinity for Alma, Rockstar, and Peaches stoperators (Fig. S2c to e). Furthermore, the Rep_Trixie_ binding affinity progressively diminishes when the substrate’s sequence is incrementally changed from a Trixie to a Peaches stoperator (Fig. S2e and f).

10.1128/mBio.00971-19.2 FIG S2 Purified Trixie immunity repressor exhibits binding affinity for cognate stoperators. (a) Coomassie-stained polyacrylamide gel reflecting purification of Rep_Trixie_, which was cloned into the pET21a vector, tagged with His, overexpressed by IPTG induction, and purified using a nickel affinity matrix. L, ladder with specific bands (in kilodaltons) labeled; U, uninduced whole-cell lysate; I, induced whole-cell lysate; P, purified protein eluted from a nickel affinity matrix. (b) Enlarged view of the capsid gene locus from a whole-genome alignment of several Cluster A phages using Phamerator. Each genome contains a predicted stoperator (open arrowhead) at the syntenic position immediately downstream of the capsid gene. (c and d) Quantification of data from electrophoretic mobility shift assays (EMSAs) (not shown) in which the binding affinity of purified Rep_Trixie_ for different stoperators at the capsid gene locus was tested. Various amounts of Rep were incubated with radiolabeled 30-bp double-stranded DNA (dsDNA) substrates derived from the phage genomes indicated at the right, with subclusters labeled. The 13-bp stoperator site is indicated by the black line. (Left) The percentage of DNA bound by Rep was measured for each Rep concentration. *K_D_* values and standard errors are indicated at the far right. (e and f) The binding affinity of Rep_Trixie_ was tested and quantified for 30-bp Peaches (“Pch.”) and negative-control (“Neg.”) substrates as well as a series of 30-bp substrates derived from the Trixie substrate containing progressive nucleotide changes within the central 13-bp stoperator site, such that the stoperator site in the final C_9_G_10_C_11_A_12_ substrate matches the stoperator site in the Peaches substrate. Download FIG S2, TIF file, 4.2 MB. Copyright © 2019 Mavrich and Hatfull. 2019 Mavrich and Hatfull This content is distributed under the terms of the Creative Commons Attribution 4.0 International license.

Taken together, these data show that these L5 clade phages have similar genome organizations and expression profiles, that the repressor system is likely the only influence on superinfection phenotypes, and that variations in the repressor and stoperator sequences are likely to contribute to differences in superinfection properties.

### L5 clade phages exhibit diverse infection phenotypes.

To determine how these diverse regulatory systems relate to superinfection immunity, we selected 19 phages from 7 subclusters across the L5 clade representing various degrees of genetic diversity based on their gene content, immunity system regulatory elements, and prophage inheritance strategies (Table 1). Lysogens were generated with each phage as well as with Dreamboat (Subcluster A1) as a heterotypic control. Superinfection immunity assays were performed against these lysogens using a variety of phages, including the parent temperate phages from which the lysogens were created, several naturally occurring L5 clade isolates that are obligately lytic derivatives of temperate parents, and several heterotypic Cluster A phages, including Peaches (Subcluster A4), Bxb1 (Subcluster A1), and Petruchio (Subcluster A1) (Table 1).

**TABLE 1:** Phages used for immunity assays

Phage	Designated subcluster	Prophage inheritance strategy^*a*^	Lys recovery^*b*^	Parent phage^*c*^	Type of mutant phage^*d*^	Mutation(s)^*e*^
Bxb1	A1	*int*	Yes	NA	NA	NA
Dreamboat	A1	*int*	Yes	NA	NA	NA
Petruchio	A1	*int*	Yes	NA	NA	NA
MissWhite	A2	*int*	No	?	NA	(Δ*rep*)
D29	A2	*int*	NA	?	NA	(*rep* Δ5′ end)
Echild	A2	*parABS*	No	NA	NA	NA
Journey13	A2	*int*	No	NA	NA	NA
Piro94	A2	*int*	No	NA	NA	NA
ArcherNM	A2	*parABS*	Yes	NA	NA	NA
Drake55	A2	*int*	Yes	NA	NA	NA
Jaan	A2	*int*	Yes	NA	NA	NA
L5	A2	*int*	Yes	NA	NA	NA
LadyBird	A2	*parABS*	Yes	NA	NA	NA
Larenn	A2	*int*	Yes	NA	NA	NA
RedRock	A2	*parABS*	Yes	NA	NA	NA
Serenity	A2	*int*	Yes	NA	NA	NA
StarStuff	A2	*int*	Yes	NA	NA	NA
Trixie	A2	*int*	Yes	NA	NA	NA
Updawg	A2	*int*	Yes	NA	NA	NA
Peaches	A4	*int*	NA	NA	NA	NA
Jeffabunny	A6	*parABS*	No	?	NA	(Δ*rep*)
DaVinci	A6	*parABS*	Yes	NA	NA	NA
Gladiator	A6	*parABS*	Yes	NA	NA	NA
Alma	A9	*parABS*	Yes	NA	NA	NA
Pioneer	A9	*parABS*	Yes	NA	NA	NA
Et2Brutus	A11	*parABS*	Yes	NA	NA	NA
Mulciber	A11	*parABS*	Yes	NA	NA	NA
DarthPhader	A12	*int*	Yes	NA	NA	NA
EagleEye	A16	*parABS*	Yes	NA	NA	NA
phiTM45	A1	*int*	NA	Bxb1	Bxb1 CRS DEM	G44351A (Rep Q138*)
phiTM1	A2	*int*	Yes	L5	BRED	44333:44334 27-bp ins (Rep-HA)
phiTM4	A2	*int*	No	phiTM1	Unintentional isolate	G43843T (gp70 A145E)
phiTM6	A2	*int*	Yes	L5	BRED	44333:44334 24-bp ins (Rep-FLAG)
phiTM33	A2	*int*	No	Che12	Unintentional isolate	Δ44705:47517 (*rep* Δ5′ end); C18749A (gp29 F223L)
phiTM41	A2	*int*	Yes	L5	Trixie Lys DEM	G50942T (gp89 F47L)
phiTM42	A2	*int*	NA	Trixie/RedRock	Trixie Lys DEM	rec Tx 44620:44630 and RR 44127:44137; rec Tx 53508:53524 and RR 53314:53330; RR Δ47296:51394; RR 45315:45316 G insertion (Rep R149fs)
phiTM43	A2	*int*	No	D29	Unintentional isolate	C25024T (gp32 P202S)
phiTM44	A2	*int*	No	D29	Unintentional isolate	C25024A (gp32 P202T); C40378T (gp59.2 sense)
phiTM38	A2	*int*	NA	phiTM44	Et2Brutus Lys DEM	C45518A (*rep* pm)
phiTM46	A6	*parABS*	NA	DaVinci	Gladiator CRS DEM	43427:43428 G insertion (Rep R52fs)
phiTM47	A6	*parABS*	NA	Gladiator	Gladiator CRS DEM	43878:43879 G insertion (Rep G135fs)
phiTM35	A9	*parABS*	NA	Pioneer	EagleEye Lys DEM	G44573T (Rep Y48*); Δ3425:5091
phiTM39	A11	*parABS*	NA	Et2Brutus	L5 Lys DEM	G44580T (Rep S102*); T7649G (Holin V9G); G50548T (gp98 sense)
phiTM40	A11	*parABS*	NA	Et2Brutus	Trixie Lys DEM	G44772A (Rep A38V); T7667G (Holin I15S)
phiTM36	A16	*parABS*	NA	EagleEye	Pioneer Lys DEM	Δ45310:48001 (Δ*rep*)

a Integrating (*int*) or extrachromosomal (*parABS*) prophage inheritance strategy.

b Lysogens (Lys) were recovered (Yes), not recovered (No), or not attempted (NA).

c Phage from which the mutant phage is derived. Natural isolates that are obligately lytic mutant derivatives of temperate phages are indicated with “?.”

d Type of mutant phage, including an unintentional isolate, a recombinant by bacteriophage recombineering of electroporated DNA (BRED), or a defense escape mutant (DEM), from either the indicated lysogen (Lys) or cloned-repressor strain (CRS).

e Mutations relative to the indicated parent phage are reported as top-strand genomic coordinates in the parent phage genome sequence from the Actinobacteriophage_1321 database and include point mutations (pm), insertions (ins) (with coordinates indicating nucleotides flanking the insertion), deletions (Δ) (with coordinates indicating the first and last nucleotides of the deleted region), and recombinations (rec) (with coordinates indicating regions of crossover within each genome). The mutational impacts on select genes and proteins (such as the *rep* locus) for mutants or natural isolates are indicated in parentheses, including sense mutations, missense mutations, nonsense mutations (*), frameshifts (fs) (with the first amino acid impacted), and complete gene deletions (Δ).

**FIG 4:**
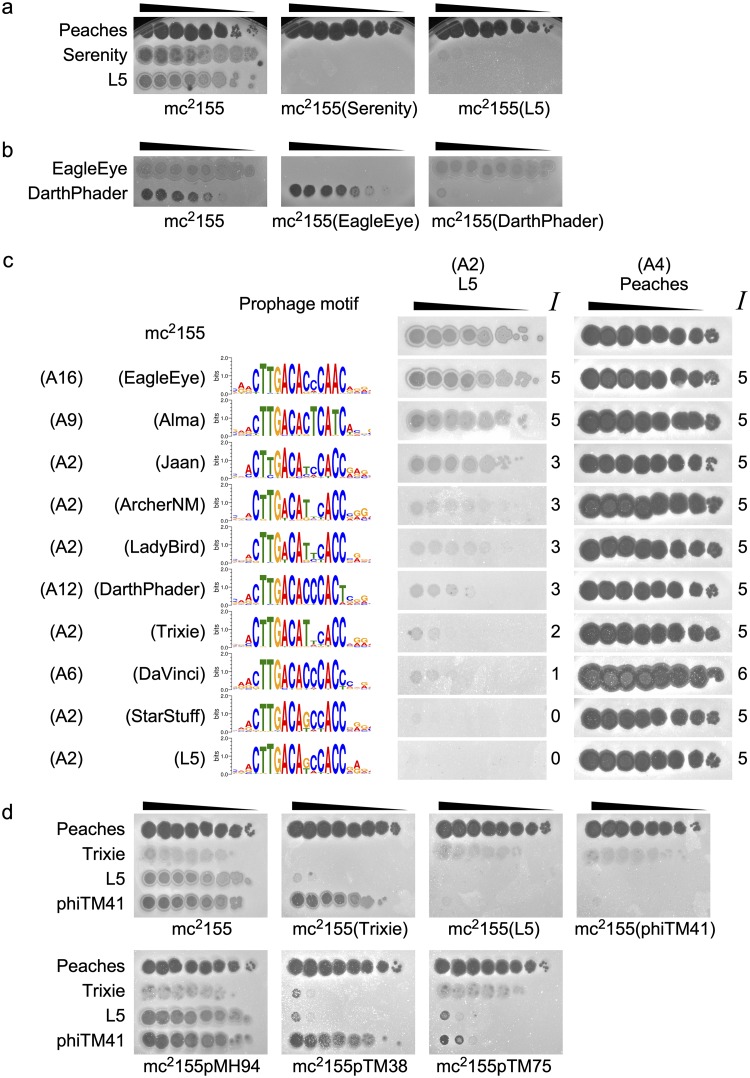
L5 clade phages exhibit diverse infection phenotypes. (a) Representative immunity assays exhibiting symmetric immunity (homoimmunity) between phages Serenity and L5. Peaches is used as a heterotypic control. Black triangles indicate 10-fold serial dilutions of the phage lysate. (b) Representative immunity assays exhibiting symmetric infection (heteroimmunity) between phages EagleEye and DarthPhader. (c) Representative immunity assays exhibiting infection phenotypes of L5 against M. smegmatis mc^2^155 and lysogens harboring prophages from the L5 clade. Peaches (Subcluster A4) is used as a heterotypic control. The subclusters and stoperator motifs for each prophage are indicated. Infection phenotypes (*Ι*) on lysogens are scored relative to the infection phenotype on mc^2^155. (d) Representative immunity assays comparing infection phenotypes of Trixie, L5, and DEM phiTM41 (a derivative of L5) against mc^2^155, lysogens (Trixie, L5, and phiTM41), and CRSs (pMH94, empty vector; pTM38, Trixie; pTM75, L5). Peaches serves as a heterotypic control.

The superinfection phenotypes are complex. Obvious examples of both homoimmunity and heteroimmunity are observed (Fig. 1 and Fig. 4a and b), but there are also numerous examples of intermediate behaviors (including partially reduced infection efficiencies and changes in plaque size and morphology). These are illustrated by infection of several different lysogens either by phage L5 (Fig. 4c) or by several other phages (Fig. S3). In some instances, plaques increase in size (Fig. S3a), and this can make it appear as though the efficiency of infection is higher on a particular lysogen than on the nonlysogenic strain (Fig. S3d). Additionally, some reciprocal infection tests do not produce symmetric phenotypes (Fig. 1). For example, an L5 lysogen is sensitive to Trixie superinfection, but a Trixie lysogen completely defends against L5 infection (Fig. 4d).

10.1128/mBio.00971-19.3 FIG S3 L5 clade phages exhibit a spectrum of infection phenotypes. Shown are data for representative immunity assays for several L5 clade phages against mc^2^155 and several lysogens as in Fig. 4c, with infection scores indicated. Download FIG S3, TIF file, 10.6 MB. Copyright © 2019 Mavrich and Hatfull. 2019 Mavrich and Hatfull This content is distributed under the terms of the Creative Commons Attribution 4.0 International license.

**FIG 5:**
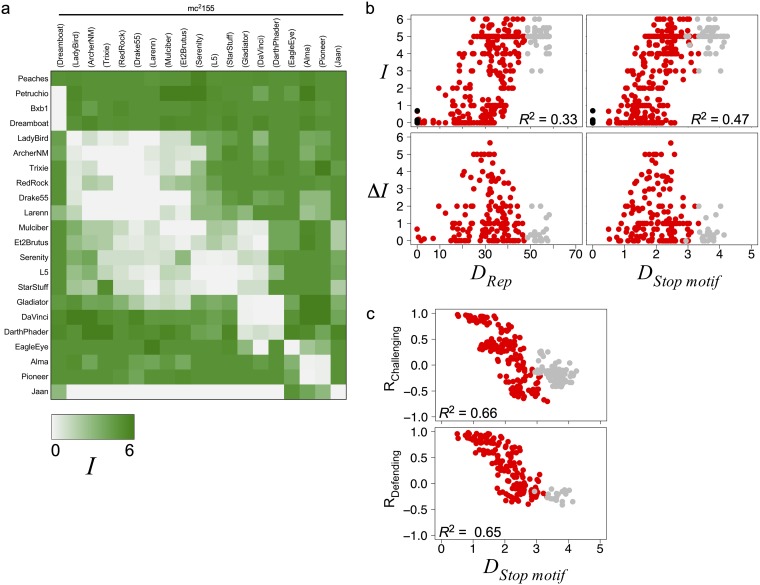
Infection patterns correlate with genetic diversity of the immunity system. (a) Heat map matrix of averaged infection scores of challenging L5 clade phages (rows) against defending L5 clade prophages (columns), where green indicates stronger infection (*Ι* = 6) and white indicates stronger defense (*Ι* = 0) (see Materials and Methods). Peaches, Dreamboat, Petruchio, and Bxb1 are used as heterotypic controls. (b) Scatterplots for all immunity assays involving an L5 clade phage with either itself (black), another L5 clade phage (red), or a non-L5-clade phage (gray) comparing Rep genetic distance (*D_Rep_*) or the stoperator motif distance (*D_Stop motif_*) with the averaged infection phenotype (*Ι*) from individual immunity assays (top) (*n* = 423) or the absolute difference in averaged infection phenotypes (Δ*Ι*) between reciprocal immunity assays (bottom) (*n* = 185). The *R*^2^ value from a linear regression of all data involving two L5 clade phages is indicated. (c) Scatterplots for pairs of phages comparing the stoperator motif distance (*D_Stop motif_*) with the correlation coefficient of the two phages’ superinfection profiles against the panel of lysogens (R_Challenging_) (top) (*n* = 225) or the superinfection profiles of phages against the two phages’ lysogens (R_Defending_) (bottom) (*n* = 171). See the Fig. 2 legend and Materials and Methods for descriptions of *D_Stop motif_* and *D_Rep_*.

To evaluate this complex set of phenotypes, we devised a scoring system (Table 2) to reflect the superinfection phenotype of each phage on each lysogen, relative to its infection of mc^2^155 (Fig. 4c) (see Materials and Methods). The infection score (*Ι*) for each assay reflects the range of phenotypes from complete immunity (in which no plaques or spots are observed [*Ι* = 0]) to complete superinfection (in which the phenotypes on the lysogen and mc^2^155 are identical [*Ι* = 5]) as well as enhanced superinfection (*Ι* = 6) (Table 2). Specific examples are illustrated in Fig. 4c and Fig. S3, and a matrix of all scores is shown in Fig. 5a. There are three key features of this matrix. First, although there are examples of complete immunity (*Ι* = 0) or complete superinfection (*Ι* = 5), there are many examples of intermediate (*Ι* = 1 to 4) or enhanced (*Ι* = 6) phenotypes (Fig. 1 and Fig. S4a). Second, the diverse phenotypes do not correlate with subcluster designations (Fig. 5a and Fig. S4b). Third, there are many examples of asymmetric phenotypes, in which the infection scores from reciprocal assays (Δ*Ι*) differ depending on which phage is superinfecting and which is defending (Fig. 1, Fig. 5a, and Fig. S4c).

**TABLE 2:** Infection scoring strategy

Score	Description of phenotype
0	No spots of lysis or plaques
1	Spots of lysis at highest 1–2 titers but no plaques
2	Superinfecting phage produces plaques with an efficiency of plating of less than ∼10^−3^–10^−4^ or spots of lysis at highest 3 titers but no plaques
3	Superinfecting phage produces plaques with an efficiency of plating from 10^−1^–10^−3^ or spots of lysis at highest 4–5 titers but no plaques
4	Superinfecting phage produces plaques with an efficiency of plating of 1, but spots/plaques exhibit increased turbidity or reduced size compared to infection of mc^2^155
5	Superinfecting phage produces plaques with an efficiency of plating of 1, and there is no phenotypic difference compared to infection of mc^2^155
6	Superinfecting phage produces plaques with an efficiency of plating of 1, but spots/plaques exhibit reduced turbidity or increased size compared to infection of mc^2^155

10.1128/mBio.00971-19.4 FIG S4 Mesoimmunity phenotypes are derived from many phages and lysogens. (a) Histograms summarizing results from all immunity assays involving L5 clade phages. Replicate infection scores for unique defending-challenging phage assay pairs were averaged and binned, and histograms reflect the percentages of defending prophages (top), challenging phages (middle), and all unique assays associated with each score (bottom). (b) Histograms as in panel A for assays involving only phages in different subclusters (top) and assays involving only phages in the same subcluster (bottom). (c) Histogram summarizing percentages of binned absolute differences in averaged infection scores (Δ*I*) between reciprocal defending-challenging phage infection tests. Download FIG S4, TIF file, 0.5 MB. Copyright © 2019 Mavrich and Hatfull. 2019 Mavrich and Hatfull This content is distributed under the terms of the Creative Commons Attribution 4.0 International license.

To investigate the causative factors of the diverse phenotypes, we compared the infection scores (*Ι*) and the reciprocity of infection scores (Δ*Ι*) to several genomic metrics. The scores correlate with changes in whole-genome gene content and nucleotide sequence (Fig. S5a). The spectrum of phenotypes occurs among phage pairs regardless of prophage inheritance genes (i.e., integration or extrachromosomal replication) (Fig. S5b and c). Interestingly, immunity scores correlate, albeit weakly, with changes in repressors or stoperator motifs (Fig. 5b) and more so than with other highly conserved genes (Fig. S5d). Also, pairwise correlations between phage defense or superinfection profiles decrease as stoperator motif distances increase (Fig. 5c). Taken together, these data suggest that the repressor-stoperator interactions play an important role in the immune phenotypes.

10.1128/mBio.00971-19.5 FIG S5 Comparison of infection phenotypes to genomic diversity. (a) Scatterplots involving an L5 clade phage with itself (black), another L5 clade phage (red), or a non-L5-clade phage (gray) comparing infection scores (*I*) (top) (*n* = 423) or absolute differences in reciprocal averaged infection scores (Δ*I*) (bottom) (*n* = 185) to whole-genome gene content (*D_GC_*) or nucleotide (*D_Nuc_*) distances. (b) Scatterplots comparing the average infection score with the stoperator motif distance (*D_Stop motif_*) between an integrated defending prophage and a challenging phage that contains an integrase gene in the same pham as the defending phage’s integrase (Same) (*n* = 65), a challenging phage that contains an integrase gene in a different pham than the defending phage’s integrase (different) (*n* = 52), or an extrachromosomal challenging phage (ParB) (*n* = 96). (c) Scatterplots comparing the infection score with the stoperator motif distance (*D_Stop motif_*) between an extrachromosomal defending prophage and a challenging phage that contains a ParB gene in the same pham as the defending phage’s ParB (Same) (*n* = 58), a challenging phage that contains a ParB gene in a different pham than the defending phage’s ParB (Different) (*n* = 42), or an integrating challenging phage (Int) (*n* = 110). (d) Scatterplots comparing the averaged infection score (*I*) (top, *n* = 423) or the absolute difference in averaged infection scores (Δ*I*) between reciprocal assays (bottom) (*n* = 185) to the genetic distance of the highly conserved Portal, DNA polymerase, EndoVII, and Cas4-family genes. All plots are formatted as described in the Fig. 5b legend. Distance metrics *D_Portal_*, *D_DNA Pol_*, *D_EndoVII_*, and *D_Cas4_* were calculated as described for *D_Rep_* in the Fig. 2 legend. See the Fig. 2 legend and Materials and Methods for a description of distances. Download FIG S5, TIF file, 1.7 MB. Copyright © 2019 Mavrich and Hatfull. 2019 Mavrich and Hatfull This content is distributed under the terms of the Creative Commons Attribution 4.0 International license.

### Diverse infection phenotypes are repressor mediated.

**FIG 6:**
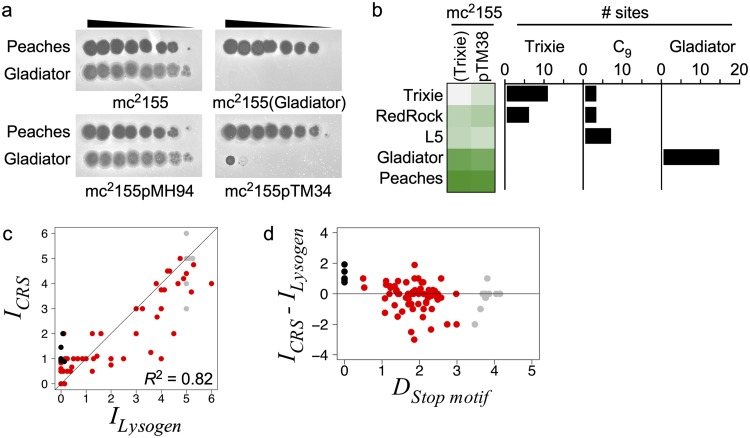
Infection patterns are repressor mediated. (a) Representative immunity assays as in Fig. 4, against mc^2^155, a lysogen (Gladiator), or CRSs (pMH94, empty vector; pTM34, Gladiator). (b, left) Heat map of infection phenotypes of Trixie, RedRock, L5, Gladiator, and Peaches against a Trixie lysogen and CRS (pTM38), as in Fig. 5a. (Right) Horizontal histogram displaying the number of 13-bp stoperator sites present in each of the challenging phage genomes that match the stoperator sites in the indicated 30-bp EMSA substrates tested for Rep_Trixie_ binding affinity in Fig. S2 in the supplemental material. (c) Scatterplot comparing superinfection scores of phages against lysogens (*Ι_Lysogen_*) and the cognate CRS (*Ι_CRS_*) (*n* = 82). The *R*^2^ value from a linear regression of all plotted data is indicated, with a color scheme as described in the Fig. 5b legend. The *y *= *x* line is plotted for reference. (d) Scatterplot comparing the change in infection scores between lysogens and cognate CRSs in panel c (*Ι_CRS_* − *Ι_Lysogen_*) and the stoperator motif distance (*D_Stop motif_*). See the Fig. 2 legend and Materials and Methods for a description of *D_Stop motif_*.

To explore further the role of the repressors, we constructed a series of recombinant strains carrying the repressor genes and upstream regulatory regions of L5, StarStuff, Trixie, Gladiator, Et2Brutus, and DaVinci (Table 3), as described previously for L5 (24), and compared their infection profiles to those of their cognate lysogens (Fig. 6). In general, there is a close correlation between infection of the “cloned-repressor strain” (CRS) and its cognate lysogen (Fig. 6a to c), consistent with the interpretation that the repressor-operator/stoperator system is the primary determinant of these diverse phenotypes. This is in contrast to phages where additional defense systems also contribute to phage susceptibilities (1, 32–34).

**TABLE 3:** Plasmids used in this study

Plasmid	Derived from:	Antibiotic marker	Description
pMH94^*a*^	NA	Kan^r^	Empty integrating vector
pTM32	pMH94	Kan^r^	Bxb1 *rep*
pTM33	pMH94	Kan^r^	Et2Brutus *rep*
pTM34	pMH94	Kan^r^	Gladiator *rep*
pTM75	pMH94	Kan^r^	L5 *rep*
pTM36	pMH94	Kan^r^	StarStuff *rep*
pTM38	pMH94	Kan^r^	Trixie *rep*
pJV44^*b*^	NA	Gent^r^	Empty extrachromosomal vector
pTM44	pJV44	Gent^r^	Empty extrachromosomal vector; ΔP*_hsp60_*
pTM48	pTM44	Gent^r^	DaVinci *rep*
pTM51	pTM44	Gent^r^	DaVinci *rep-73*

a See reference 55.

b See reference 57.

Although the CRS and lysogen infection scores generally correlate, there are two types of notable departures. In general, a CRS confers weaker immunity than the analogous lysogen against the homotypic superinfecting phage. For example, Gladiator infection of its CRS forms clearings or tiny plaques at high titers, even though the lysogen shows complete immunity (Fig. 6a). This was also reported previously for L5 (24). In contrast, some CRSs confer stronger immunity than the analogous lysogen (Fig. 6c), with the most discrepant phenotypes occurring at greater genetic distances of the stoperator motifs (Fig. 6d). The most notable difference between a Cluster A lysogen and a cognate CRS is the presence of as many as 30 stoperator sites. Although the roles of these sites during superinfection immunity are unclear, the relative affinities of the repressors for the stoperator and operator sites of both the lysogen and the infecting phage may be important. We note that for DaVinci, a CRS containing a larger DNA segment from the repressor locus restores homotypic immunity to that observed in the lysogen, which may be due to either low-level expression of the three genes downstream of the repressor, stabilized expression of the repressor itself, or the presence of an additional stoperator (Table 3 and Fig. S6).

10.1128/mBio.00971-19.6 FIG S6 Extended DaVinci *rep-73* construct enhances immunity. (a) Enlarged view of the DaVinci immunity repressor locus with the indicated *rep* and *rep-73* regions cloned into pTM48 and pTM51, respectively. Open arrowheads indicate stoperator sites. (b) Representative immunity assays comparing infection phenotypes of Gladiator, DEM phiTM47 (derivative of Gladiator), DaVinci, DEM phiTM46 (derivative of DaVinci), phiTM33 (derivative of Che12), EagleEye, and Bxb1 against mc^2^155, a DaVinci lysogen, and CRSs (pTM44, empty vector; pTM48, DaVinci *rep*; pTM51, DaVinci *rep-73*). Bxb1 is used as a heterotypic control. Download FIG S6, TIF file, 3.5 MB. Copyright © 2019 Mavrich and Hatfull. 2019 Mavrich and Hatfull This content is distributed under the terms of the Creative Commons Attribution 4.0 International license.

### Isolation and characterization of defense escape mutants.

In general, the L5 clade phages described here show strong immunity to themselves. Virulent mutants that escape homotypic immunity are not observed and likely arise only at very low frequencies, similar to phages λ (35), P22 (13), and P1 (14). However, among infections of mesotypic lysogens and CRSs, it is common to observe reduced efficiency of plating and individual mutant plaques at high titers (Table S1). These potentially represent mutational pathways to escape immunity and evolve new immune specificities. We thus isolated, sequenced, and characterized nine defense escape mutants (DEMs) that escape defense from six different lysogens or CRSs (Table 1).

10.1128/mBio.00971-19.9 TABLE S1 Processed infection data for all immunity assays performed. Each row indicates a unique infection assay involving a lysogen or CRS. Whole-genome nucleotide distance, whole-genome gene content distance, repressor genetic distance, and stoperator motif distance between the challenging phage and the resident prophage in the lysogen or the prophage that corresponds to the CRS are indicated. The number of assay replicates, the averaged infection score, as well as the minimum and maximum infection scores from replicates are indicated. Download Table S1, XLSX file, 0.1 MB. Copyright © 2019 Mavrich and Hatfull. 2019 Mavrich and Hatfull This content is distributed under the terms of the Creative Commons Attribution 4.0 International license.

**FIG 7:**
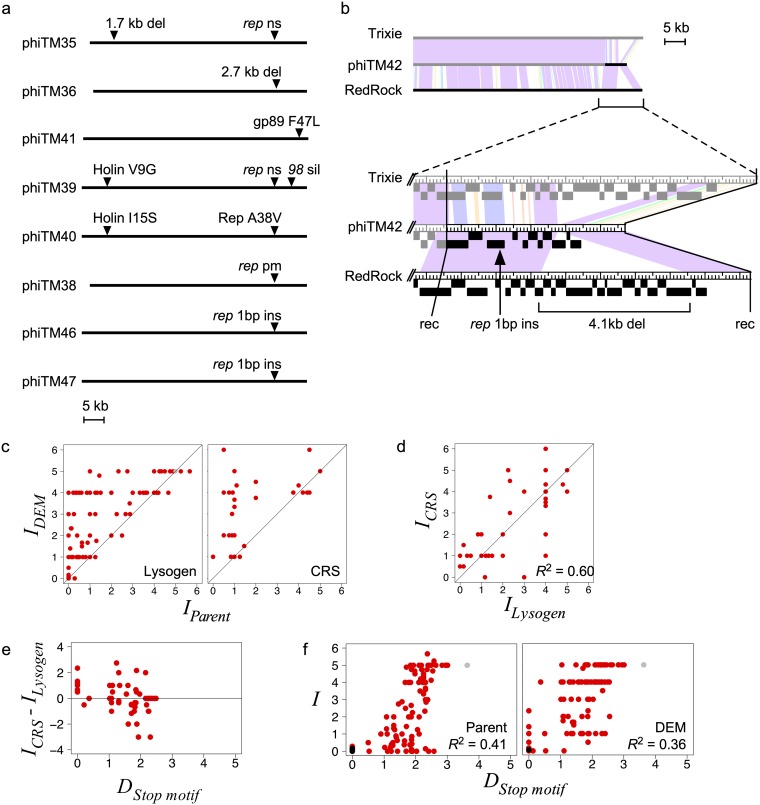
Characterization of defense escape mutant infection profiles. (a) Genome maps of several DEMs that have escaped immunity from either a lysogen or CRS (Table 1). Arrowheads indicate mutations. ns, nonsense; sil, silent; ins, insertion; del, deletion; pm, point mutation. (b, top) Whole-genome alignment of RedRock (black), Trixie (gray), and DEM phiTM42 (black and gray) using Phamerator. The color spectrum between genomes indicates sequence similarity (violet, significant similarity; white, no similarity). (Bottom) Enlarged view of the right genome termini, indicating the four mutations present in phiTM42 relative to Trixie and RedRock. rec, recombination point. (c) Scatterplots comparing infection scores of DEMs (*Ι_DEM_*) and their parent phages (*Ι_Parent_*) against lysogens (left) (*n* = 124) or CRSs (right) (*n* = 32), formatted as in Fig. 5b. (d and e) Scatterplots as in Fig. 6c and d comparing infections of DEMs against lysogens and the analogous CRSs. (*n* = 57). (f) Scatterplots comparing infection scores to stoperator motif distances (*D_Stop motif_*) for DEMs and their parent phages against lysogens, formatted as in Fig. 5b. (*n* = 124). See the Fig. 2 legend and Materials and Methods for a description of *D_Stop motif_*.

The DEMs have acquired different types of mutations (Table 1 and Fig. 7a and b). An L5 mutant, phiTM41, escapes a Trixie lysogen after acquiring a single missense mutation in the first coding sequence downstream of P_left_, gene *89* (Fig. 4d and Fig. 7a). More substantial deletions are incurred by Pioneer and EagleEye mutants, phiTM35 and phiTM36, to escape each other’s lysogen (Fig. 7a and Fig. S7a). The most dramatic mutation is observed in phiTM42, isolated from a RedRock infection of a Trixie lysogen (Fig. 7b). This DEM is a recombinant hybrid of the two phages, in which Trixie has lost the rightmost ∼10 kb of its genome (including P_left_ and *rep*) and has acquired the analogous locus from RedRock via two recombination events. Over 4 kb of the RedRock fragment has been deleted, and *rep* has acquired a 1-bp insertion. Unlike lambdoid virulent mutants (7), all DEMs (except for phiTM41) contain a mutation at the *rep* locus, and none of them involve mutations within operators (Fig. 7a and b and Fig. S7b). Thus, they may escape immunity by utilizing different pathways compared to lambdoid phages.

10.1128/mBio.00971-19.7 FIG S7 Summary of mutations present in defense escape mutants. (a) Immunity assays of Pioneer, DEM phiTM35 (derivative of Pioneer), EagleEye, and DEM phiTM36 (derivative of EagleEye) on mc^2^155, an EagleEye lysogen, and a Pioneer lysogen. (b) Codon alignment of repressors from L5 clade phages using PRANK. Nucleotide conservation is indicated below. A dashed line separates predicted N-terminal and C-terminal regions. The position of the DNA-binding helix-turn-helix domain is indicated by a thin black bar at the top. Thick black bars above the alignment indicate the repressor coding sequences in several DEMs or obligately lytic mutant isolates, highlighting mutations or truncations due to missense mutations, nonsense mutations (*), frameshifts (fs), or deletions. The black arrowhead indicates the position of the A38V missense mutation in phiTM40. phiTM33 and D29 are obligately lytic mutant isolates with N-terminally truncated repressors. phiTM44, as a derivative of D29, also contains an N-terminally truncated repressor. phiTM38, a derivative of phiTM44, has acquired a point mutation that introduces a nonsense mutation if the truncated *rep* gene is translated, and the amino acid is numbered according to the full-length Rep sequence in StarStuff, its nearest temperate relative. See Table 1 for more details. Download FIG S7, TIF file, 1.4 MB. Copyright © 2019 Mavrich and Hatfull. 2019 Mavrich and Hatfull This content is distributed under the terms of the Creative Commons Attribution 4.0 International license.

### Defense escape mutants exhibit different degrees of virulence.

We next examined how the escape mutations impact virulence against other related systems. We compared the infection strengths between each DEM and parent phage across a panel of strains. DEMs nearly always exhibit infection strengths equal to or greater than those of their parent phages on both lysogens and CRSs (Fig. 7c). Similar to naturally occurring temperate phages, DEMs occasionally exhibit increased infection on lysogens compared to CRSs (Fig. 7d and e). However, although DEMs have escaped one immunity system, they are not able to escape all immunity systems (Fig. 7f). Instead, different degrees of virulence are observed, and we highlight these distinctions below with several examples.

**FIG 8:**
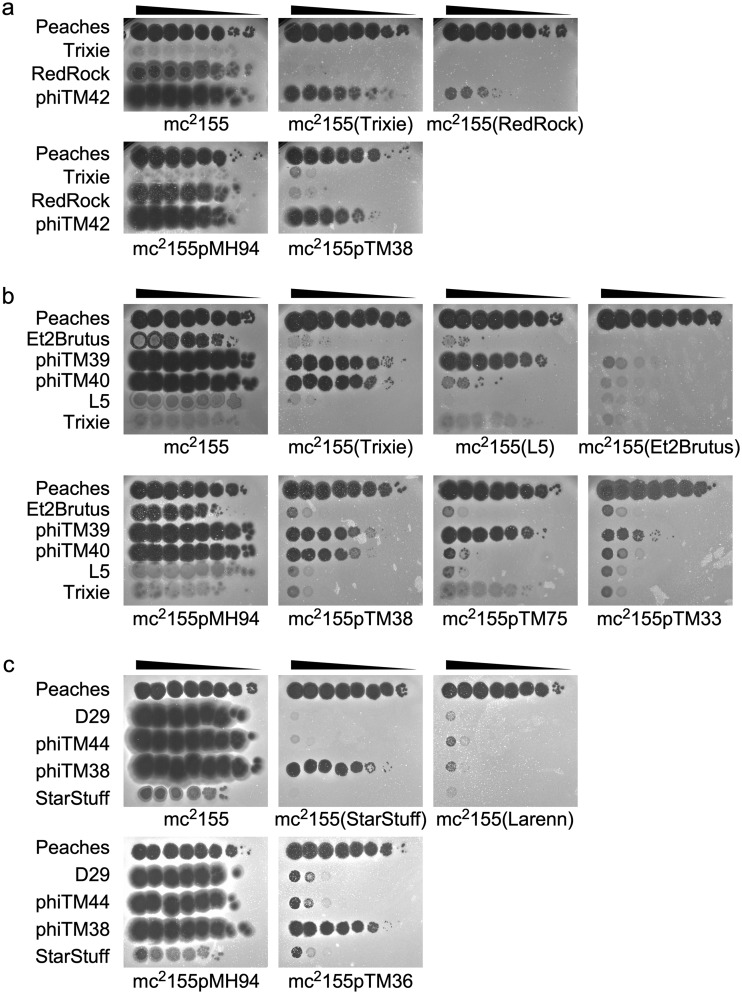
Escape mutants exhibit various degrees of virulence. (a) Representative immunity assays comparing infection phenotypes of Trixie, RedRock, and DEM phiTM42 (derivative of Trixie and RedRock) against mc^2^155, lysogens (Trixie and RedRock), and CRSs (pMH94, empty vector; pTM38, Trixie). (b) Representative immunity assays comparing infection phenotypes of Et2Brutus and DEMs phiTM39 and phiTM40 (derivatives of Et2Brutus) against mc^2^155, lysogens (Trixie, L5, and Et2Brutus), and CRSs (pMH94, empty vector; pTM38, Trixie; pTM75, L5; pTM33, Et2Brutus). L5 and Trixie phages serve as negative controls for lysogens and CRSs. (c) Representative immunity assays involving D29, phiTM44 (derivative of D29), and DEM phiTM38 (derivative of phiTM44) against mc^2^155, lysogens (StarStuff and Larenn), and CRSs (pMH94, empty vector; pTM36, StarStuff). StarStuff serves as a negative control for lysogens and CRSs.

The expanded host range (or “virulence specificity”) is variable, as observed with phiTM41 and phiTM42. The mutation in phiTM41 confers narrow mesotypic virulence, as it does not impact infection of any lysogens other than Trixie (Fig. 4d and see Fig. 10c). phiTM41 is the only DEM with no mutation in *rep*, its plaque morphology is not substantially impacted, and it remains temperate (Fig. S8f). phiTM41 lysogens exhibit the same defense profile as an L5 lysogen (Fig. 4d and see Fig. 10c), indicating that the missense mutation in gp89 (which has no known function) abolishes the asymmetric infection observed between Trixie and L5 without altering other infection or defense capabilities. In contrast, phiTM42 is obligately lytic and exhibits broad homotypic and mesotypic virulence; it escapes immunity from both parent phages (Trixie and RedRock) and a Trixie CRS (Fig. 8a) as well as every other lysogen tested (Fig. S8a). However, phiTM42 plaques are noticeably smaller on Trixie and RedRock lysogens than on mc^2^155, suggesting that some degree of superinfection inhibition remains (Fig. 8a).

10.1128/mBio.00971-19.8 FIG S8 Infection profiles of defense escape mutants and engineered L5 mutants. (a, b, d, and e) Heat maps of infection phenotypes as in Fig. 5a comparing the infection profiles of several DEMs with those of their parent phages against lysogens and CRSs (pTM33, Et2Brutus; pTM36, StarStuff; pTM38, Trixie; pTM75, L5). Columns are ordered by increasing infection strength of the parent phage. phiTM42 is a derivative of Trixie and RedRock, phiTM39 and phiTM40 are derivatives of Et2Brutus, phiTM46 is a derivative of DaVinci, phiTM47 is a derivative of Gladiator, Jeffabunny is an obligately lytic mutant isolate, phiTM44 is a derivative of D29, and phiTM38 is a derivative of phiTM44 (Table 1). (c) Phamerator whole-genome alignment of Subcluster A6 phages, as in Fig. 7b. (f) Comparison of plaque morphologies from wild-type L5 and several L5 derivatives. (g) Scatterplots as in Fig. 5b comparing infection phenotypes of L5 and phiTM41 (*n* = 19), phiTM6 (*n* = 22), or phiTM1 (*n* = 17) against lysogens. The *y *= *x* line is plotted for reference. (h) Scatterplots comparing infection phenotypes of phages against an L5 lysogen and phiTM41 (*n* = 33), phiTM6 (*n* = 42), or phiTM1 (*n* = 34) lysogens. Download FIG S8, TIF file, 3.4 MB. Copyright © 2019 Mavrich and Hatfull. 2019 Mavrich and Hatfull This content is distributed under the terms of the Creative Commons Attribution 4.0 International license.

Mutations may confer nuanced virulence specificities, as observed with Et2Brutus escape mutants phiTM39 and phiTM40 isolated on L5 and Trixie lysogens, respectively. phiTM40 has an A38V substitution in the *rep* DNA-binding domain, whereas phiTM39 has a nonsense mutation at codon 102 of *rep* (although both have additional mutations which could influence their phenotypes [Table 1]). Both phages infect the CRS strains cognate to the lysogens on which they were isolated (Fig. 8b), but phiTM39 has a more relaxed specificity than phiTM40, exhibiting stronger infection of several strains (Fig. S8b), including an L5 lysogen (Fig. 8b). It even produces tiny, faint plaques on the homotypic Et2Brutus lysogen (Fig. 8b). Thus, the type of repressor mutation alters the superinfecting phage’s behavior, depending on the nature of the lysogen being infected.

An additional example of the complexities of the escape phenotypes is illustrated by DEMs phiTM46 and phiTM47 (Table 1), which are derivatives of the closely related phages DaVinci and Gladiator, respectively, and have similar stoperator motifs (Fig. 2e and Fig. S8c). Both phiTM46 and phiTM47 have a 1-bp insertion within *rep* that presumably generates a truncated, inactive product (Fig. 7a and Fig. S7b). However, the infection properties of these DEMs depart from those of their parents in distinct ways (compare infection profiles in Fig. S8d), suggesting that features other than the repressor *per se* influence these behaviors. One plausible explanation is that variation among the individual stoperator sites plays an important role.

An especially striking phenotype is exhibited by the DEM phiTM38. phiTM38 is a derivative of phage D29, an obligately lytic phage due to a deletion of the 5′ end of the repressor gene (Fig. S7b) (31). Unlike its parent, phiTM38 efficiently escapes immunity of the homotypic StarStuff lysogen and CRS (Fig. 8c) as well as the mesotypic Et2Brutus lysogen on which it was isolated (Table 1). In contrast, its infection profile on other lysogens is unchanged (Fig. S8e), as illustrated with the mesotypic Larenn lysogen (Fig. 8c). Surprisingly, the single point mutation in phiTM38 maps to the remaining 3′ segment of the inactive repressor gene (Fig. S7b). The simplest explanation is that this influences the expression of the downstream genes during superinfection and that these also play a role in the complex immunity relationships.

### Both the *rep* DNA-binding domain and C terminus confer immune specificity.

**FIG 9:**
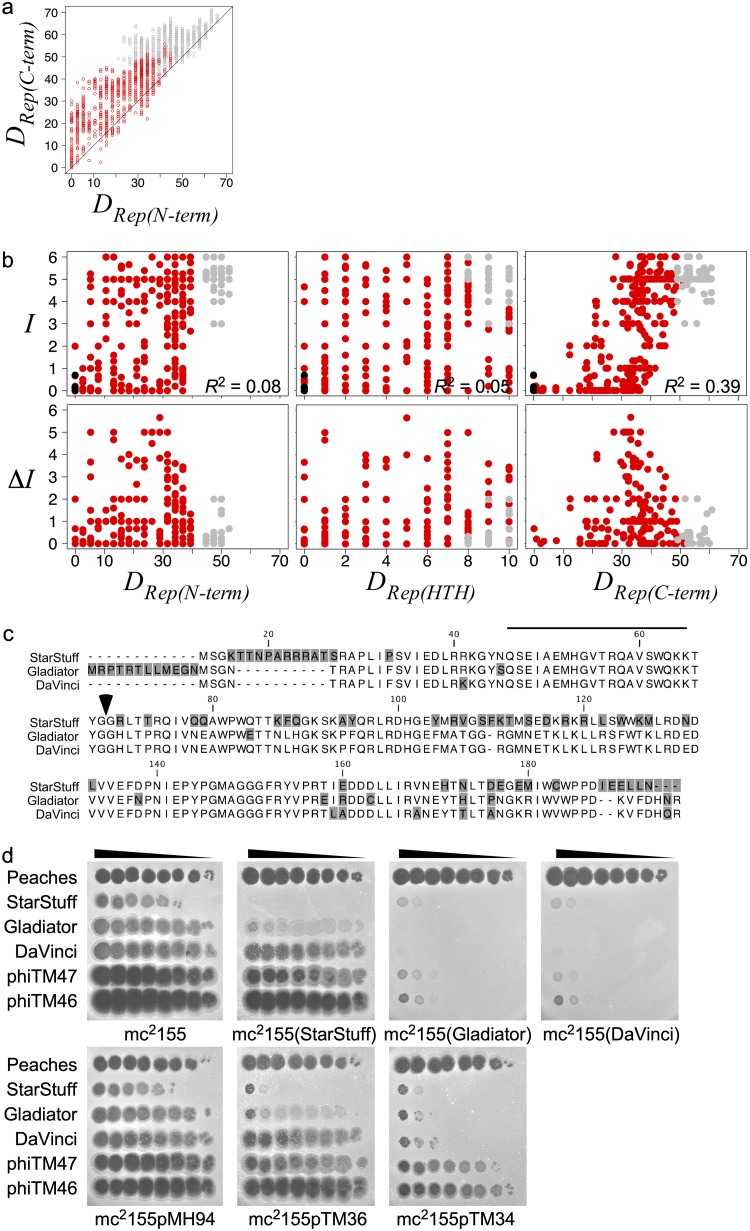
The repressor C terminus impacts immune specificity. (a) Scatterplot comparing repressor N-terminal region [*D_Rep_*_(_*_N-term_*_)_] and C-terminal region [*D_Rep_*_(_*_C-term_*_)_] genetic distances for 87 L5 clade phages. (b) Scatterplots comparing the averaged infection score (Ι) (top) (*n* = 423) or the absolute difference in averaged infection scores (ΔΙ) between reciprocal assays (bottom) (*n* = 185) to the genetic distance of the repressor N-terminal region or C-terminal region or the Hamming distance of the predicted helix-turn-helix domain [*D_Rep_*_(_*_HTH_*_)_], formatted as in Fig. 5b. (c) Alignment of StarStuff, Gladiator, and DaVinci Rep homologs, with the helix-turn-helix domain indicated by a black bar, the N-terminal and C-terminal regions demarcated by an arrow, and amino acid variants shaded in gray. (d) Immunity assays involving StarStuff, Gladiator, DaVinci, DEM phiTM47 (derivative of Gladiator), and DEM phiTM46 (derivative of DaVinci) against mc^2^155, lysogens (StarStuff, Gladiator, and DaVinci), and CRSs (pMH94, empty vector; pTM36, StarStuff; pTM34, Gladiator). Peaches serves as a heterotypic control. Distance metrics *D_Rep_*_(_*_N-term_*_)_ and *D_Rep_*_(_*_C-term_*_)_ were calculated as described in the Fig. 2 legend for *D_Rep_*. *D_Rep_*_(_*_HTH_*_)_ indicates the number of amino acids that are different. See the Fig. 2 legend and Materials and Methods for a description of distances.

The immunity repressors of lambdoid phages contain a helix-turn-helix domain near the N terminus responsible for DNA binding and a C-terminal domain responsible for dimerization that impacts sequence specificity (36–38). Cluster A Rep similarly has an N-terminal helix-turn-helix DNA-binding domain (16, 24) and a distinct C-terminal region (27). Nucleotide sequence alignment of the immunity repressors from the L5 clade suggests that these two regions are under markedly different evolutionary pressures (Fig. S7b), with greater diversity in the C-terminal region than in the N-terminal region (Fig. 9a). Surprisingly, differences in the divergent C-terminal regions correlate with the immunity phenotypes rather than differences in the N-terminal DNA-binding domains (Fig. 9b). For instance, Rep_StarStuff_, Rep_Gladiator_, and Rep_DaVinci_ differ primarily in their C-terminal regions (and share an identical helix-turn-helix domain) (Fig. 9c), but their stoperator motifs are distinct (Fig. 2e and Fig. 4c), and they exhibit asymmetric superinfection phenotypes on lysogens and CRSs (Fig. 9d). Thus, both the N-terminal DNA-binding domain and the C-terminal region influence Cluster A Rep immune specificity.

### An engineered L5 mutant exhibits narrow homotypic virulence.

**FIG 10:**
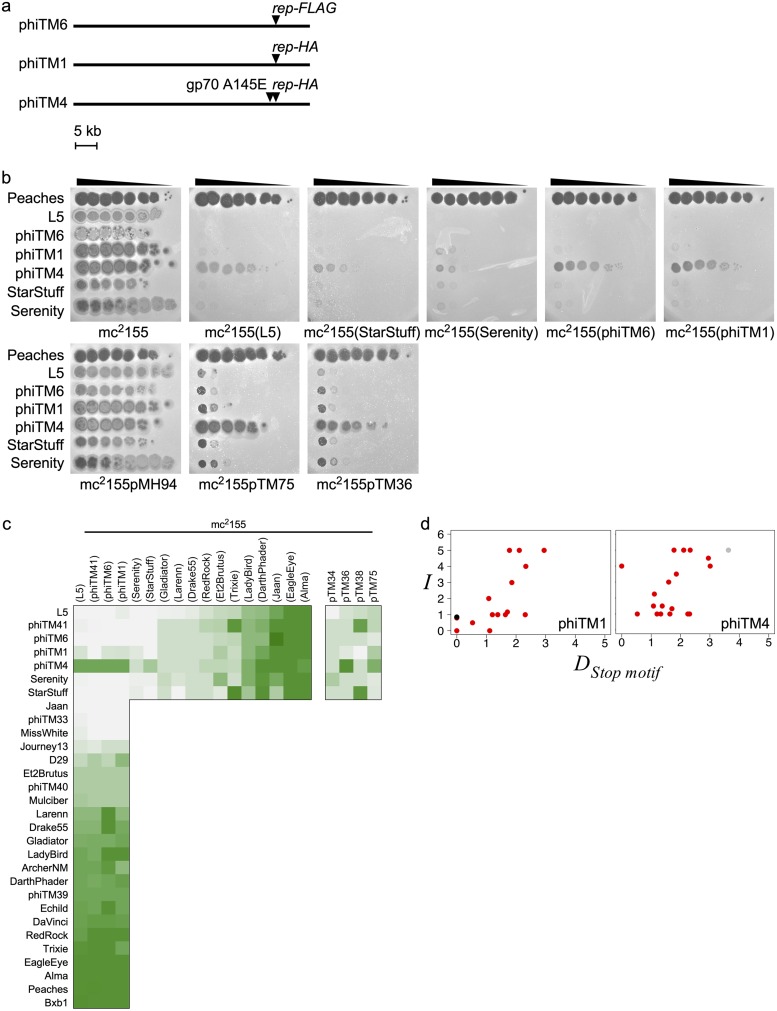
An engineered L5 mutant exhibits narrow homotypic virulence. (a) Genome maps of L5 and derivative mutants indicating the engineered mutations present in phiTM6 (FLAG-tagged *rep*) and phiTM1 (HA-tagged *rep*) and the unintentional mutation acquired in phiTM4 (derivative of phiTM1) (Table 1). (b) Representative immunity assays comparing infection phenotypes of L5 and several derivatives (phiTM6, phiTM1, and phiTM4) against mc^2^155, lysogens (L5, StarStuff, Serenity, phiTM6, and phiTM1), and CRSs (pMH94, empty vector; pTM75, L5; pTM36, StarStuff). Peaches serves as a heterotypic control, and Serenity and StarStuff serve as negative controls for lysogens and CRSs. (c) Heat map of infection phenotypes as in Fig. 5a, comparing infection profiles of phages against L5, phiTM41, phiTM1, and phiTM6 lysogens and infection profiles of L5, phiTM41, phiTM1, phiTM4, phiTM6, Serenity, and StarStuff against several lysogens and CRSs (pTM75, L5; pTM36, StarStuff; pTM38, Trixie; pTM34, Gladiator). Rows are ordered by increasing infection strength on an L5 lysogen, and columns are ordered by increasing L5 infection strength. (d) Scatterplots comparing infection scores to the stoperator motif distance (*D_Stop motif_*) of phiTM1 (*n* = 17) and phiTM4 (*n* = 22) infections against lysogens, formatted as in Fig. 5b. See the Fig. 2 legend and Materials and Methods for a description of *D_Stop motif_*.

As part of a study of repressor function, we constructed two derivatives of phage L5 in which either a FLAG (phiTM6) or a hemagglutinin (HA) (phiTM1) tag is added to the C terminus of the repressor (Fig. 10a). These derivatives exhibit only subtle differences in plaque morphology (Fig. S8f), and they retain the ability to lysogenize. Their infection and immunity profiles are similar to those of their L5 parent (Fig. 10b and c and Fig. S8g and h). During purification of a phiTM1 lysogen, a homotypic virulent mutant derivative, phiTM4, was isolated. phiTM4 has acquired a point mutation within gene *70*, a gene of unknown function located immediately downstream of *rep* (Table 1 and Fig. 10a). The point mutation changes the last amino acid in gp70, but it also occurs within a stoperator site, although this site deviates from the consensus and is not bound by Rep *in vitro* (9). phiTM4 superinfects lysogens of all L5 derivatives as well as a StarStuff lysogen and L5 and StarStuff cognate CRSs (Fig. 10b and c). However, it remains unable to superinfect lysogens generated from other phages, including its closest relative, Serenity (Fig. 10b to d). The single point mutation confers the narrowest homotypic virulence observed: phiTM4 escapes homotypic immunity but remains subject to closely related immunity systems.

### Et2Brutus relatives exhibit nonlinear immune specificity evolution.

**FIG 11:**
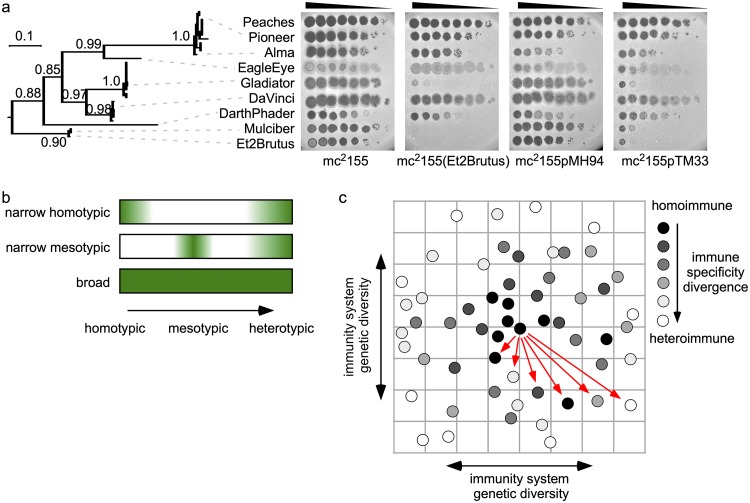
Evolution of immunity systems and virulence. (a, left) Phylogeny of immunity repressors from 33 phages representing Subclusters A9, A16, A6, A12, and A11 constructed using maximum likelihood based on codon alignment (see Materials and Methods). Branch support values reflect data from an approximate likelihood ratio test. (Right) Immunity assays involving representative phages from across the phylogeny (dashed lines) against mc^2^155, an Et2Brutus lysogen, and CRSs (pMH94, empty vector; pTM33, Et2Brutus). Peaches serves as a heterotypic control and is not represented in the phylogeny. (b) Diagram summarizing three different types of virulence observed among DEMs, defined by their ability to superinfect lysogens carrying prophages across a genetic spectrum of immune specificities, using a color spectrum as in Fig. 5a. (c) Model of immune specificity evolution. Circles represent phage genomes on a theoretical landscape of genetic diversity, and distance from the reference phage (centered circle) represents increasing genetic diversity of immunity system regulatory elements. Circles are shaded based on reciprocal superinfection immunity phenotypes relative to the reference phage and range from homoimmunity (black) to heteroimmunity (white). Genetic relationships to selected phages across the genetic landscape (red arrows) highlight that homoimmunity, mesoimmunity, and heteroimmunity may emerge multiple times as immunity systems diverge.

To understand the evolutionary history of immune specificities, we compared superinfection phenotypes within a phylogenetic context. A phylogeny was constructed from all *rep* nucleotide sequences in the L5 clade, and a subclade representing several subclusters and exhibiting a robust tree topology was evaluated (Fig. 11a). An Et2Brutus lysogen and CRS exhibit immunity to Et2Brutus and Mulciber (both Subcluster A11). They exhibit sensitivity to other phages across this subclade, including DaVinci (Subcluster A6), but they are immune to Gladiator (also Subcluster A6). These immunity patterns are not congruent with the phylogeny, suggesting that immune specificities have switched more than once (Fig. 11a). Escape from Et2Brutus immunity may have emerged in a distant ancestor near the root of the tree, followed by immunity reemerging among some Subcluster A6 phages. Alternatively, escape from immunity may have independently emerged multiple times across this subclade.

### Function and evolution of other Cluster A phages.

There are over 200 Cluster A phages outside the L5-related clade. The majority of these phages also contain syntenically positioned immunity repressors and predicted stoperators (data not shown). A Bxb1 (Subcluster A1) CRS, constructed analogously to the L5 clade CRSs, is immune to Bxb1 superinfection, and a Bxb1 mutant (phiTM45) escapes this CRS after acquiring a single nonsense mutation in *rep* (Table 1 and Table S1). Therefore, the function and evolution of the immunity system within L5 clade phages may extend to all Cluster A phages.

## DISCUSSION

Classical models of superinfection, immunity, virulence, and the evolution of new immune specificities were primarily developed with limited collections of coliphages related to λ, P22, and P1 (7, 14). However, the genetic diversity among Cluster A phages illustrates how an immunity system can gradually evolve into homologous, mesotypic circuits with regulatory elements that exhibit a spectrum of interactions (Fig. 1). Interactions between mesotypic systems generate superinfection immunity patterns that are not necessarily binary or symmetric. In this more complex genetic environment, virulence and specificity can be shaped by both homotypic and mesotypic phages (Fig. 1). For instance, the mutations acquired in phiTM38 and phiTM39 conferring escape from mesotypic immunity systems also enable homotypic virulence. Additionally, there are different degrees of virulence within a mesoimmunity group. Both phiTM38 and phiTM4 exhibit narrow homotypic virulence, with little to no impact on escape from mesotypic systems (Fig. 11b). phiTM39 exhibits weak homotypic virulence but enhanced mesotypic virulence. phiTM41 exhibits narrow mesotypic virulence, while phiTM42 exhibits broad homotypic and mesotypic virulence (Fig. 11b). Thus, evolutionary divergence between repressor-mediated immune specificities may not be a linear trajectory toward heteroimmunity. Instead, genetic interactions with homotypic and mesotypic phages may result in a meandering evolutionary path in which homoimmunity, mesoimmunity, and heteroimmunity emerge more than once (Fig. 11c).

Homotypic virulence can occur by disrupting interactions between the repressor and cognate binding sites, as observed for λ and P22 (7), or by disrupting interactions between factors at secondary immunity loci, as seen in P1 and P7 (8). In contrast to these systems, the majority of Cluster A DEMs have acquired mutations that inactivate the repressor, and none of them (with the possible exception of phiTM4) contain a mutation within an operator or stoperator. Rep is the only identified transcriptional regulator involved in initiating and maintaining lysogeny for Cluster A phages (24). However, we do not fully understand how the Cluster A immunity system functions, and other factors may be involved, similar to systems in P1, P7, and N15. The diverse types of mutations in phiTM39, phiTM40, phiTM41, and phiTM42 conferring escape from a Trixie lysogen may be targeting different aspects of the immunity system. phiTM39 and phiTM40 may escape with a modified Rep, while phiTM41 escapes with a modified gp89. The dramatic recombination in phiTM42 enabling escape from all lysogens may combine discordant regulatory elements in Trixie and RedRock that no individual prophage is able to properly regulate at the same time. Meanwhile, the mutation in phiTM4 appears to disrupt a very specific interaction present within L5, derivatives of L5, and StarStuff, such that it does not disrupt interactions within other prophages.

The diverse range of infection phenotypes observed on lysogens or CRSs could be caused by several factors related to Rep expression and specificity. Immunity asymmetry may be caused by repressors with sufficiently similar, but distinct, binding affinities or specificities (39), combined with subtle differences in the sequences and positions of stoperators, such that only one of the repressors is able to prevent lytic gene expression in both genomes. Gradual fading of spot dilutions could reflect weak affinity or specificity of the prophage’s Rep for stoperators in the superinfecting phage genome that is sufficient to interfere with, but not completely defend against, superinfection and lytic growth, resulting in smaller or more turbid plaques and spots. Enlarged spot dilutions or plaques (suggesting enhanced infection) could reflect negative interactions between the prophage’s and the superinfecting phage’s regulatory elements that reduce the efficiency of lysogenization without inhibiting lytic growth. Differences in CRS and lysogen immunity may result in differences in Rep expression levels or in the number of available stoperators. Other factors may be involved in this immunity system that we have not yet identified, and the evolution of these factors may also contribute to the diverse infection phenotypes.

Mesoimmunity groups are likely to be common in nature. There are several groups of actinobacteriophages infecting *Gordonia*, *Rhodococcus*, and *Streptomyces* hosts that harbor immunity systems similar to those of the Cluster A mycobacteriophages (40, 41). Additionally, we note that there are examples of asymmetric and incomplete infection among phages related to λ (42) and P2 (15). The complex pathways of immunity evolution are likely common features of temperate phages although readily apparent only when comparing large groups of related temperate phages known to infect a common host bacterium.

## MATERIALS AND METHODS

### Phamerator database construction.

The database Actinobacteriophage_1321 was created using Phamerator (43), consisting of 1,305 manually annotated genomes of actinobacteriophages isolated from the environment and 16 engineered or isolated mutants as described below. Genes are grouped into phamilies (“phams”) based on amino acid sequence similarity using kClust implemented in the Phamerator pipeline (23). The database is available online (http://phamerator.webfactional.com/databases_Hatfull).

### Identification and analysis of stoperator sequences.

Stoperator sequences were automatically identified in all Cluster A genomes using MEME (44), using the following parameters: site distribution of any number of repetitions, maximum of 2 motifs, motif length of 12 to 16 bp, consisting of 10 to 50 sites, and derived from both strands. The motif that most closely resembled empirically determined L5 and Bxb1 stoperator sites was selected. All sites representing each motif were converted to the sense strand and manually aligned in Excel. Motif logos representing the aligned sequences were created with WebLogo (45). Stoperator sequences were compared in R using the Biostrings and TFBSTools packages (46). Position weight matrices (PWMs) of the core 13-bp sequence were created using the PFMatrix and toPWM functions, using the log2probratio method and default values for background and pseudocount settings. Pairwise PWM-normalized Euclidean distances were computed using the PWMSimilarity function, and larger distances represent more dissimilar PWMs (46, 47). Similar to whole-genome distance metrics (described below), stoperator motif distances can be computed between all L5 clade phages. Stoperators were determined to be oriented in the direction of transcription (syn oriented) if they were located on the top strand to the left of the genome center or on the bottom strand to the right of the genome center. The center of the genome was defined as the coordinates of the integrase (for integrating phages) or *parA* (for extrachromosomal phages) gene. To generate genomic distributions of stoperators in L5 clade phages, coordinates of all stoperators in each phage were adjusted relative to the coordinates of the genomic feature of interest in that specific phage, and histograms were created using adjusted coordinates for all L5 clade phages.

### Computation of whole-genome distances.

Pairwise nucleotide similarity and gene content dissimilarity between all phage genomes were computed, as previously described (48). For pairs of phages, gene content dissimilarity ranges from 0 (all gene phams are identical) to 1 (no gene phams are identical), and nucleotide distance ranges from 0 (identical sequence) to 0.5 (unrelated sequence).

### Genetic distance of specific Cluster A genes.

Amino acid sequences for 336 full-length homologs of the Cluster A immunity repressor present in the database (represented by phams 3247, 38916, and 38877) were aligned using MAFFT (49). The alignment was manually trimmed at the N terminus in SeaView (50) and split into N-terminal and C-terminal regions as previously reported (27). Uncorrected distances between taxa in the full-length, N-terminal, and C-terminal alignments were computed using the EMBOSS distmat tool with no gap weight and reported as a normalized distance reflecting the number of substitutions per 100 amino acids (https://www.ebi.ac.uk/Tools/emboss/). The 20-amino-acid helix-turn-helix domain was identified in all taxa from the MAFFT alignment based on previous reports (16, 26). Uncorrected distances for full-length proteins of 311 homologs of the Cas4-family gene (pham 29663), 306 homologs of Endonuclease VII (EndoVII) (pham 39443), 311 homologs of DNA polymerase (pham 39585), and 311 homologs of Portal (pham 38438) genes present in Cluster A phages were computed in the same way. Hamming distances between helix-turn-helix domains were computed using the stringdist R package. Unlike whole-genome distances and stoperator motif distances, gene-specific distances are limited to phages that carry a homolog of the gene of interest.

### Repressor nucleotide alignment and phylogeny.

Nucleotide sequences for 79 immunity repressors from L5 clade phages were aligned by codon using webPRANK (51), and a phylogenetic tree was constructed using maximum likelihood in SeaView (50) and annotated using Evolview (52).

### Preparation of phage lysates and lysogens.

A diverse set of phages was selected for immunity assays, representing multiple subclusters, utilizing different prophage inheritance strategies (integration or extrachromosomal partitioning), and carrying complete or mutant repressor genes (Table 1). All phages used for immunity assays were plaque purified at least twice. Lysates were expanded for one round from a plaque pick by plating phage with mc^2^155, incubation at 37°C for 24 to 36 h, incubation with 5 ml phage buffer at room temperature for 4 to 5 h, and filtering with a 0.22-μm filter. Lysates were confirmed to have the expected phage by PCR using primers that amplify near the right genome terminus, in which there is substantial sequence diversity among Cluster A phages, to generate phage-specific amplicons. Lysogens for many purified phages were created by spotting high-titer phage lysates on a lawn of mc^2^155, picking cells from the center of the spot after 3 to 7 days, and performing colony purification at least two times. Strains were confirmed as lysogens by PCR using the same primers as those used for lysate confirmation, by verifying that cells exhibit spontaneous phage release when spotted onto a lawn of mc^2^155, and by verifying that the strain is immune to infection from the parent phage. Lysogens for phages Echild (30), Journey13, and Piro94 could not be generated (Table 1). Lysogenization of some phages was not tested (Table 1).

### RNA-seq.

Strand-specific transcription profiles of Et2Brutus, Gladiator, and Trixie lysogens were measured as previously described (1) and viewed using Integrated Genomics Viewer (IGV) (53).

### Repressor overexpression and EMSAs.

Rep_Trixie_ was amplified from the Trixie genome (coordinates 45599 to 46174) with primers oTM13 and oTM14 and cloned into the expression vector pET21a using the NdeI and HindIII sites to create the plasmid pTM1, which carries Rep_Trixie_ C-terminally tagged with His and a short linker (KLAAALEHHHHHH). pTM1 was transformed into NEB5α cells. Sequence-verified plasmid constructs were transformed into BL21 Star(DE3) cells, and single colonies were grown in LB medium supplemented with carbenicillin. Repressor expression was induced with 1 mM isopropyl-β-d-thiogalactopyranoside (IPTG) for 3 h, and cells were lysed by resuspension in lysis buffer (50 mM Tris [pH 8.0], 300 mM NaCl, 10% glycerol), treatment with 1 mg/ml lysozyme for 30 min on ice, and light sonication (54). C-terminally His-tagged Rep_Trixie_ was purified using a nickel-nitrilotriacetic acid (NTA) matrix, dialyzed overnight with storage buffer (50 mM Tris [pH 8.0], 150 mM NaCl, 0.1 mM EDTA, 0.1 mM dithiothreitol [DTT], 50% glycerol), and quantified at ∼1 mg/ml using a NanoDrop instrument (Thermo Fisher). DNA substrates for electrophoretic mobility shift assays (EMSAs) were designed to be 30 bp long, consisting of a 13-bp stoperator sequence flanked by 8 to 9 bp of sequence. Complementary 30-bp oligonucleotides were synthesized, radiolabeled at the 5′ end with γ-^32^P, and annealed (54). Oligonucleotides for each substrate in Fig. S2 in the supplemental materials are as follows: oTM21 and oTM22 for Alma, oTM23 and oTM24 for Gladiator, oTM17 and oTM18 for Peaches, oTM31 and oTM32 for RedRock, oTM19 and oTM20 for Rockstar, oTM33 and oTM34 for Trixie, oTM29 and oTM30 for the L5 gene *31* negative control, oTM43 and oTM44 for C_9_, oTM41 and oTM42 for C_9_G_10_, oTM39 and oTM40 for C_9_C_11_, oTM37 and oTM38 for C_9_A_12_, oTM49 and oTM50 for C_9_G_10_C_11_, oTM47 and oTM48 for C_9_G_10_A_12_, oTM45 and oTM46 for C_9_C_11_A_12_, and oTM35 and oTM36 for C_9_G_10_C_11_A_12_ (Table S2). The sequences of the 30-bp substrates to test syntenic stoperator sites, including the L5 gene *31* negative control, are derived directly from the genome sequence. For the 30-bp substrates in which the Trixie stoperator site is progressively converted to a Peaches stoperator site, the variable 13-bp sequence is flanked by invariable 8 to 9 bp derived from the Trixie substrate. EMSAs were performed with serially diluted Rep, which was electrophoresed on an 8% polyacrylamide gel and imaged, as previously described (54). The *K_D_* for each substrate was calculated with nonlinear regression in Prism software using the one-site-specific binding option and least-squares fit.

10.1128/mBio.00971-19.10 TABLE S2 Oligonucleotides used to clone and purify Rep_Trixie_, to generate EMSA substrates, to generate cloned-repressor strains, and to C-terminally tag the L5 repressor. Download Table S2, XLSX file, 0.02 MB. Copyright © 2019 Mavrich and Hatfull. 2019 Mavrich and Hatfull This content is distributed under the terms of the Creative Commons Attribution 4.0 International license.

### Construction of cloned-repressor strains.

The immunity repressors from several Cluster A phages were cloned into the integrating vector pMH94 (55). The ∼1- to 1.5-kb locus, consisting of *rep*, its promoter, and part of the flanking upstream and downstream genes, was amplified by PCR in phages L5 (coordinates 44037 to 45330 using primers oTM194 and oTM195), StarStuff (coordinates 45039 to 46286 using primers oTM196 and oTM197), Et2Brutus (coordinates 44069 to 45220 using primers oTM190 and oTM191), Trixie (coordinates 45266 to 46542 using primers oTM198 and oTM199), Gladiator (coordinates 43468 to 44632 using primers oTM192 and oTM193), and Bxb1 (coordinates 43962 to 45171 using primers oTM188 and oTM189) (Table S2). Primers contained partial homology to pMH94 flanking the XbaI site. Amplicons were purified with the NucleoSpin PCR cleanup kit, and pMH94 was linearized with XbaI and purified with the NucleoSpin gel cleanup kit. The linearized vector and amplicon were ligated using Gibson assembly (56) and transformed into NEB5α cells. The following plasmids were constructed: pTM75 (L5 *rep*), pTM36 (StarStuff *rep*), pTM33 (Et2Brutus *rep*), pTM38 (Trixie *rep*), pTM34 (Gladiator *rep*), and pTM32 (Bxb1 *rep*) (Table 3). Sequence-verified constructs were transformed into electrocompetent M. smegmatis mc^2^155. Positive transformants were selected using LB medium supplemented with kanamycin and clonally purified.

The immunity repressor from DaVinci was cloned into the extrachromosomal multicopy vector pJV44 (57). The locus was amplified by PCR using primers containing XbaI and HindIII sites. For constructs containing only *rep*, analogous to the integrated repressor constructs described above, a segment from DaVinci (coordinates 42748 to 43932) was amplified using primers oTM257 and oTM265. For the construct containing the extended repressor locus (from *rep* to gene *73*), a segment from DaVinci (coordinates 41377 to 43932) was amplified using primers oTM257 and oTM258. pJV44 and the amplicons were digested with XbaI and HindIII, gel purified and cleaned up using the NucleoSpin gel extraction kit, ligated with T4 DNA ligase, and transformed into NEB5α cells. Since this cloning strategy removes the *hsp60* promoter in pJV44, a religated vector backbone that lacks the *hsp60* promoter was constructed as an empty vector control using an amplicon directly generated from self-amplifying primers (oTM266 and oTM267). The following plasmids were constructed: pTM44 (empty vector), pTM48 (DaVinci *rep*), and pTM51 (DaVinci *rep-73*) (Table 3). Sequence-verified plasmid constructs were transformed into electrocompetent mc^2^155 cells, and positive transformants were selected using Middlebrook 7H10 medium supplemented with gentamicin and clonally purified.

### L5 repressor tagging.

Rep_L5_ was C-terminally tagged *in vivo* with either a 27-bp HA (phiTM1 [TACCCATACGACGTCCCAGACTACGCT]) or a 24-bp FLAG (phiTM6 [GACTACAAGGACGACGATGACAAG]) (58) sequence using recombineering with an L5 lysogen, similar to previous reports (59). The FLAG oligonucleotide (oTM51) and HA oligonucleotide (oTM52) were PCR amplified using primers oTM53 and oTM54 to create ∼200-bp recombineering substrates that overlap the 3′ end of gene *71* and that contain the tag sequence (Table S2). Amplicons were purified using the GeneJet PCR purification kit, and the DNA was cotransformed with pJV44 into electrocompetent mc^2^155(L5)pJV53, as previously described (59). Successful pJV44 transformants were selected on Middlebrook 7H10 medium supplemented with gentamicin, and successful L5 recombinants were subsequently identified by PCR. Positive recombinants were clonally purified and patched onto a lawn of mc^2^155, and spontaneously released phages were picked and plaque purified. From one of the HA-tagged recombinant phage picks, a spontaneous mutation was acquired (phiTM4).

### Immunity assays.

Fresh 10-fold serial dilutions of each phage lysate were generated using phage buffer (10 mM Tris [pH 7.5], 10 mM MgSO_4_, 68 mM NaCl, 1 mM CaCl_2_), and 3 μl of each dilution was spotted onto a top agar layer of the indicated strain. For immunity tests involving lysogens, strains were plated in Middlebrook 7H9 top agar on Middlebrook 7H10 medium. For immunity tests involving strains carrying pMH94-derived cloned-repressor constructs, strains were plated in Middlebrook 7H9 plus kanamycin top agar on Mycobacteria 7H11 plus kanamycin medium. For immunity tests involving strains carrying pJV44-derived cloned-repressor constructs, strains were plated in Middlebrook 7H9 plus gentamicin top agar on Middlebrook 7H10 plus gentamicin medium. Lysates were always spotted onto an accompanying nonlysogen or empty vector control strain (mc^2^155, mc^2^155pMH94, or mc^2^155pTM44) for reference. Plates were incubated at 37°C for 3 days and photographed with ImageLab using a 1.5- to 2.0-s exposure. Individual assays were quantitatively scored by comparing the qualitative infection phenotypes of the phage on the strain of interest to those for the control strain, including efficiency of plating, turbidity, the presence of plaques, and plaque size (Table S1). Results were processed in R using custom scripts. More than 3,000 immunity assays were performed and manually scored, representing 1,050 unique comparisons, 239 reciprocal comparisons, and 164 lysogen-CRS paired comparisons. The bacterial densities for each culture used are approximately 1 × 10^9^ to 4 × 10^9^ CFU per 1 ml of culture. We estimate that spots from the highest titers of phage represent phage infections that occur (on average) with a multiplicity of infection (MOI) of between 2 and 20. Therefore, the infection phenotypes for the majority of spot dilutions represent phage infections at an MOI of less than 1.

### Isolation of defense escape mutants.

Mutant phages able to escape prophage or cloned-repressor defense were isolated by picking plaques from immunity assays in which the challenging phage exhibits a substantial reduction in efficiency of plating, performing plaque purification at least twice on mc^2^155, and confirming the ability to infect the original strain (Table 1). DNA was extracted from both the DEM and parent phage lysates and sequenced as previously described (1). Mutations were identified by whole-genome alignment. In some cases, the parent phage genome contained a mutation(s) relative to the published sequence. Only mutations that are present in the DEM compared to the parent are reported.

### R data analysis.

Infection data were analyzed and visualized in RStudio using custom scripts with the reshape2 and stringdist packages (Table S1). More than 65% of unique comparisons were measured with two or more replicate assays, and replicate infection scores were averaged. Although the infection score can vary between replicates, more than 80% of comparisons with two or more replicates exhibit a range of infection scores lower than 2. All *R*^2^ correlations between genetic elements and immunity phenotypes were determined with linear regression only using intra-L5 clade comparisons, unless otherwise indicated, using the lm function.

### Data availability.

Raw fastq data have been deposited in the Gene Expression Omnibus (GEO) under accession number GSE123612. The R code used for data analyses is available upon request.
